# Synergistic fields: Unveiling the potential win-win relationship between esports performance and traditional sports participation

**DOI:** 10.1371/journal.pone.0305880

**Published:** 2024-08-12

**Authors:** Di Tang, Ruisi Ma, Peichi Chung, Wai-keung Ho, Kim-wai Raymond Sum

**Affiliations:** 1 The Department of Sports Science and Physical Education, The Chinese University of Hong Kong, Hong Kong, China; 2 School of Physical Education, Jinan University, Guangzhou, China; 3 The Department of Cultural and Religious Studies, The Chinese University of Hong Kong, Hong Kong, China; IBS Hyderabad: ICFAI Business School, INDIA

## Abstract

This cross-sectional study investigated the association between participation in traditional sports and esports performance, analyzing data from 1,549 survey respondents, with a specific focus on 617 individuals assessed for their esports capabilities. The analysis suggests that participation in traditional sports is associated with enhanced performance in first-person shooters. However, no similar association was observed in multiplayer online battle arena games across various platforms. Furthermore, although no substantial link was found between overall gameplay duration and esports performance for the majority of the games examined, time spent playing Honor of Kings was significantly associated with improved in-game rankings, likely due to its unique matchmaking and rating system. The findings also indicate that participants from different categories of traditional sports show no significant differences in esports performance, suggesting similar physical and athletic requirements across these sports. This underscores the necessity for further exploration and methodological refinement to investigate the associations between specific types of physical exercise and enhancements in esports performance. Additionally, esports participants demonstrated higher levels of engagement in traditional sports compared to their non-esports counterparts, suggesting potential reciprocal benefits between esports and physical exercise. Future research should further investigate these mutual advantages. Conducting additional rigorous empirical research to substantiate these associations is essential for the sustainable development of esports.

## Introduction

Esports commonly refers to competitive video gaming [1–3]. According to one of the most cited definitions, esports is "a form of sports where the primary aspects of the sport are facilitated by electronic systems; the input of players and teams, as well as the output of the esports system, are mediated by human-computer interfaces" [4,5]. An increasing number of countries, such as Brazil, Bulgaria, China, Italy, Korea, Russia, and the US, have already considered esports as an official sport [6], and it is already under consideration in about 40 countries [7]. At the 2023 Asian Games, esports was included as a formal medal event [8]. Governments and academic institutions are paying more attention to esports. The valuation of the esports sector reached $USD 24.9 billion as of 2020, with forecasts suggesting the audience could expand to 650 million by 2025, growing at an annual rate of 8.1% [9–11]. The Hong Kong government has dedicated 100 million HKD to enhance its esports sector, with initiatives aimed at supporting venue infrastructure, technology, and talent cultivation [12]. Research conducted by the Hong Kong Federation of Youth Groups involving 1,400 young individuals between 15 and 29 years old showed that within a six-month timeframe, 38.3% had viewed esports events, and 13.4% had actively participated [13]. However, despite the significant development of esports as an industry, the theoretical foundation and scientific research in esports lag behind its practical advancements. Between 2002 and March 2018, fewer than 200 articles on esports were published across four major databases. Moreover, there is scant research from research institutions or groups in China despite the fact that China and Asia are critical regions for the development of esports [14–16].

While the number of esports studies has increased significantly since 2018, research on esports has primarily focused on media, informatics, and business, with only a minimal number of studies from a sports science perspective [14,17]. As a recognized competitive sport, it is crucial to develop scientific training models for esports and to establish metrics for performance enhancement. Currently, literature on these aspects remains sparse [18]. Recent studies, including a performance structure proposed by Nagorsky and Wiemeyer, highlight the need for prioritizing and developing specific skills and training domains in esports, akin to those valued in traditional sports [19]. Sanz-Matesanz et al. conducted a scoping review to examine the physical and psychological factors influencing esports performance, underscoring the urgent need to develop effective interventions to boost players’ skills and their overall well-being [18]. Additionally, numerous studies have explored the link between mental and cognitive skills and esports performance, focusing on reaction time, stress-coping strategies, executive functions, and personality traits. However, the causal relationships and underlying mechanisms are not yet clear, although the established correlations provide useful insights for future training models and intervention strategies [20–25].

In many studies, physical exercise has shown positive impacts on executive functions, reaction time, communication skills, and stress-coping strategies. For instance, Colcombe and Kramer demonstrated that aerobic fitness training enhances cognitive functions [26,27]. A meta-analysis confirmed significant improvements in reaction time, independent of exercise duration, intensity, or the age of the subjects [28]. Cognitive functions improve through enhanced blood flow, central nervous system activity, and neurotransmitter engagement [29–31]. Frith and Loprinzi found that lower extremity muscular strength was associated with enhanced executive cognitive skills [32]. Additionally, physical exercise positively influences communication skills [33,34].

The suggested structure of esports performance indicates that skills benefiting from physical exercise are also critical to esports performance [19]. Physical ability and effort are significant in esports, influencing competition outcomes [3,19]. Despite these findings, more empirical research and long-term evidence are needed to definitively link physical exercise with esports performance or to demonstrate the impact of physical exercise on such performance. Current research often relies on indirect indicators, such as cognitive test data, to assess esports performance. A direct, comprehensive indicator or measurable model is still required.

Physical exercise is also an effective intervention for managing chronic pain severity with minimal side effects [35,36]. Chronic pain, including neck, wrist, hand, and back pain, frequently affects esports players, often due to high-intensity training or muscle overuse [23,37,38]. Therefore, investigating the relationship between physical exercise and esports, or the impact of physical exercise on enhancing performance, as well as its role in rehabilitation and injury prevention, is undoubtedly crucial for a scientific esports practice and modality. However, the current body of related literature remains insufficient [18].

To address these research gaps, this study is designed as a cross-sectional study to explore the relationship between esports performance and traditional sports participation, to provide foundational evidence for a longitudinal study on the effects of physical training on esports, and to conduct a preliminary investigation for the future development of more rational and scientific esports training models.

## Literature review

### Esports performance and measurement

To date, no uniform or consensus measurement method or system exists for evaluating performance in esports. In the esports domain, it is recognized that performance encompasses not only physical capabilities, such as upper limb stability, hand-eye coordination, and manual dexterity, but also cognitive and psychological skills and qualities, including stress resilience, reactive capabilities, problem-solving abilities, and multitasking proficiency [19,39]. Consequently, these attributes are reflected in the measures used to assess esports performance.

In existing literature related to electronic sports, the most widely used methods for testing esports performance can be summarized into the following three categories:

The first type of evaluation method relies on players’ rankings or scores within the specific esports titles they participate in [18,20,40]. This approach provides an overall assessment of a player’s comprehensive abilities, from technical skills to psychological robustness, thus offering a holistic view of an individual’s esports literacy [18]. However, scoring systems vary significantly across different esports titles, and only those players who actively engage in ranked matches have a corresponding rank. Those who do not participate in ranked play lack an equivalent evaluation. This method presents challenges in assessing a broader range of esports player abilities and performances. Additionally, factors such as the matchmaking mechanism, the amount of time invested by the player, and the structure of the player’s team can significantly influence in-game ranks and levels, raising questions about whether these ranks truly reflect an individual’s skill level [41].

The second type of measurement involves analyzing players’ performance data from specific game sessions or performance demonstrated by avatars in custom-designed scenarios [40,42–44]. Such tests can offer more direct insights into a player’s proficiency in particular operations or key in-game techniques. However, due to the relatively short duration of a single game or task and the reduced emphasis on team coordination and competitive play against real opponents, these tests may not accurately reflect the psychological demands of actual competition, thus failing to provide a comprehensive assessment.

The final category encompasses more indirect methods, such as psychological tests, reaction time assessments, and the application of validated tests known to relate to abilities pertinent to esports [29,44–47]. These measures have been shown to correlate with esports performance and include evaluations of reaction time, decision-making, and concentration. While the tests employed in this approach are often more scientifically robust, having been validated through numerous studies, they are indirect measures of ability and may not precisely or objectively equate to actual esports performance. In existing research, cognitive tests have not demonstrated sufficient discriminative power to differentiate levels of esports performance within certain games [45,46].

### Esports performance and physical exercise

According to the theoretical models of esports performance and training developed by Nagorsky and Wiemeyer, many skills associated with traditional sports performance play a significant role in the realm of esports [19]. They identified numerous similarities in abilities, such as tactical-cognitive abilities, coordination skills, and psychic or mental abilities, within existing theoretical models of training and performance in sports and game competencies. Based on these findings, they integrated an esports performance model. This integration illustrates the potential relationships between physical training and esports performance. However, more empirical evidence is still required to validate this theoretical model. Additionally, recent studies indicate that after aerobic exercise, players show enhanced performance in esports tasks designed by researchers [40,43,47]. Physical exercise appears to have a warm-up effect on esports competition. However, the long-term influence of these exercise-induced effects on improving esports performance still requires further investigation.

Additionally, DiFrancisco-Donoghue observed that intermittent six-minute walks during esports gameplay can similarly enhance performance [47]. These interventional studies suggest that the integration of physical activity and traditional sports training methods into esports presents considerable potential. Moreover, the well-documented benefits of physical sports for health could provide a mitigating effect against the health risks associated with sedentary behaviors prevalent in esports. How long-term physical exercise habits or training should be incorporated into esports training and the daily lives of esports players becomes an important area for future exploration. This current study represents a preliminary investigation of the relationship between long-term sports habits and esports performance.

### Research questions

1. Is there a difference in esports performance between participants who are actively involved in traditional sports and those who do not engage in such activities?
2. Is there a difference in esports performance among participants engaged in different categories of traditional sports?
3. Is there a difference in participation in traditional sports between esports players and non-esports players?

## Materials and methods

### Research objective

The objective of this study is to explore the relationship between esports performance and traditional sports participation.

### Participants

Participants were recruited through social media and university student emails in Hong Kong from September 2022 to February 2023. An online survey was sent to the students’ emails to collect relevant data. A total of 1,549 responses were received, of which 633 participants were identified as esports players. Of these, 617 players were included in the esports performance statistical analysis; the remaining players reported that they had never participated in ranking games, or that the esports games they played did not have a standardized and uniform ranking system (e.g., FIFA), which resulted in missing data on their esports performance.

This study categorized esports participants as mainstream esports gamers, engaging in various genres, including multiplayer online battle arenas (MOBA), fighting games, real-time strategy (RTS) games, strategy card games, sports simulation games, and shooting games. MOBA is characterized by two teams competing to destroy each other’s base, where teamwork and coordination are crucial for success [48]. In RTS games, players command a multitude of units and structures from a top-down perspective, issuing real-time orders to gather resources, construct infrastructure and armies, and ultimately defeat enemy forces [49].

### Measurement

An online questionnaire was used to collect the data from the participants. The questionnaire was divided into three sections: basic information (age, sex, etc.), gaming information (ranking in games, game genres, playing frequency, and gameplay time, etc.), and traditional sports participation (frequency, duration, sports type).

A participant’s in-game ranking was used as an indicator of esports performance in this game. The in-game ranking is an indicator of a player’s level of performance based on their win rate and in-game performance data (e.g., kills, deaths, the rank of opponents defeated, etc.) through participation in ranking matches in different esports games [20,50,51]. In many leading esports, player rankings are determined using a Match Making Rating (MMR). It aims to equalize the skill levels among competitors or teams. Under normal circumstances, we assume that players or teams with higher MMR will defeat those with MMR. However, under this system, if a player with a lower MMR defeats a higher MMR player, they will earn better points [52].

In the context of esports, taking League of Legends (LOL) as an example, the ranking system is organized from lowest to highest as follows: Iron, Bronze, Silver, Gold, Platinum, Diamond, Master, Grandmaster, and Challenger. Each of these ranks was assigned a specific value at the outset for the purposes of evaluation and comparison. Starting from 1, each rank was given a score, and one additional score was given for each increase in ranking. Since different esports games have different ranking criteria and different classifications, this study discussed the different gaming categories of esports players separately.

### Statistical analysis

Descriptive information was calculated on all variables included in the present study. The statistical analysis in this study was conducted using IBM SPSS. Analysis of covariance (ANCOVA), Gamma test, Chi-square test, and Mann-Whitney U test were conducted to analyze the data. Differences were considered statistically significant if the p-value was < .05. G*power software was used to evaluate the sample size for the analysis in each game genre.

### Ethics approval and consent to participate

This study was approved by the Research Ethics Committee of The Chinese University of Hong Kong (January 2022; Reference No. SBRE‐21‐0432) (see S1 and S2 Files). The study complies with the relevant rules and regulations of research ethics. Written informed consent was obtained from all the participants before they participated in the research. This form was provided beforehand and requires consent to indicate that they fully understand and agree to the terms and conditions of the survey and research. Only after signing the informed consent form online can they proceed to take the official survey. The questionnaire does not involve any sensitive information that makes participants identified. All collected data were saved on a personal computer with the investigator’s password only.

## Results

The age of the participants included in the esports performance analysis is 18.78 ± 1.51. There are 368 male participants and 249 female participants. A total of 404 participants reported participating in traditional sports, while 213 did not. Table 1 shows the types of esports games played and the number of years of gaming experience among the participants.

**Table 1:** Esports performance and gaming experience of players in different genres.

		LOL	Dota2	HOK/AOV	Hearthstone	Street Fighter	PUBG	Total
Performance	Mean (SD)	4.49 (1.58)	3.83 (2.04)	5.64 (1.48)	4.42 (1.46)	3.4 (1.67)	4.18 (1.68)	
Rank range	Low–High	1–9	1–8	1–7	1–6	1–11	1–8	
Experience	<1 year	2	1	5	2	0	2	12
	1–3 years	12	0	24	0	0	18	54
	3.1–5 years	18	0	64	3	0	30	115
	5.1–7 years	27	1	50	3	2	22	105
	7.1–9 years	26	0	34	9	0	13	82
	>9 years	87	4	86	16	3	53	249
Participants		172	6	263	33	5	138	617

(LOL: League of Legends; Dota2: Defense of the Ancients 2; PUBG: Player Unknown’s Battlegrounds; HOK/AOV: Honor of Kings/Arena of Valor).

According to the sample size, only participants in LOL, Honor of Kings/Garena Arena of Valor, and PUBG were considered suitable for the following analysis. These three esports games represent mainstream games in the esports industry: MOBA (PC), MOBA (Mobile), and FPS (First-Person Shooter).

### Traditional sports participation and esports performance

Since in-game ranking might be correlated with the duration of esports involvement and the number of years of participation among players in this study varies, the number of years a player has been involved in the game was added to the analysis as a covariate. ANCOVA was used to determine if there was a significant difference in rankings between two groups of participants (those physically active and those not).

As shown in Table 2, no significant difference was observed between the exercise and non-exercise groups in LOL (*p*>0.05). The duration of time spent in LOL was not associated with esports performance. In HOK/AOV, no significant difference was found between the two groups (*p*>0.05), and players with different years of gaming experience showed significant differences in ranking (*p*<0.05). The ranking in PUBG between the two groups was statistically significant (*p*<0.05). Physically active participants had higher rankings in PUBG. However, the years of a player’s participation in the game were not significantly related to their esports performance. Longer gaming experience did not contribute significantly to a player’s performance in PUBG.

**Table 2:** Esports performance between sports group and non-sports group.

	Participants	Performance (95% CI)	F	*p*
LOL				
	Traditional Sports Players	4.39±1.60 (4.10–4.67)	1.74	0.19
	Non-Traditional Sports Players	4.74±1.55 (4.30–5.16)		
	Year (participation in esports)		0.134	0.715
HOK/AOV				
	Traditional Sports Players	5.69±1.49 (5.46–5.90)	0.29	0.59
	Non-Traditional Sports Players	5.56±1.46 (5.28–5.88)		
	Year		5.989	*0.02
PUBG				
	Traditional Sports Players	4.46±1.53 (4.11–4.83)	6.788	**0.008
	Non-Traditional Sports Players	3.73±1.78 (3.29–4.16)		
	Year		0.41	0.52

* *p*<0.05

** *p*<0.01

*** *p*<0.001.

#### Sports types and esports performance

A total of 32 types of traditional sports were identified: baseball, bowling, squash, jogging, cycling, frisbee, cycling, basketball, volleyball, etc. They are divided into different groups of sports types to see if the sports type has an association with esports performance: combative and non-combative sports, collective sports, and individual sports.

As shown in Tables 3 and 4, no significant difference in the esports performance was found between combative and non-combative sports or team-based and individual sports. Similarly, the years of a player’s participation in Honor of Kings were significantly related to their final game rank, which was not observed in PUBG and LOL. Meanwhile, we also collected data on the frequency and duration of traditional sports among the athletes and conducted a correlation analysis with the players’ gaming performance. However, no significant correlation was found.

**Table 3:** Esports performance between combative and non-combative sports group.

	Sports Type	Performance (95% CI)	F	*p*
LOL				
	Combative	4.39±1.60 (4.10–4.67)	0.46	0.46
	Non-combative	4.74±1.55 (4.30–5.16)		
	Year		2.56	0.11
HOK/AOV				
	Combative	5.90±1.47 (5.47–6.21)	0.76	0.38
	Non-combative	5.56±1.46 (5.35–5.92)		
	Year		7.79	**0.006
PUBG				
	Combative	4.46±1.53 (4.11–4.83)	1.68	0.20
	Non-combative	3.73±1.78 (3.29–4.16)		
	Year		0.01	0.95

* *p*<0.05

** *p*<0.01

*** *p*<0.001.

**Table 4:** Esports performance between team-based and individual sports group.

	Sports Type	Performance (95% CI)	F	*p*
LOL				
	Team-based	4.62±1.47 (4.15–5.08)	1.469	0.22
	Individual	4.22±1.67 (3.85–4.62)		
	Year		6.11	0.44
HOK/AOV				
	Team-based	5.93±1.41 (5.50–6.25)	1.43	0.29
	Individual	5.59±1.53 (5.34–5.90)		
	Year		7.87	**0.006
PUBG				
	Team-based	4.86±1.53 (4.30–5.43)	2.20	0.14
	Individual	4.33±1.52 (3.91–4.76)		
	Year		0.15	0.90

* *p*<0.05

** *p*<0.01

*** *p*<0.001.

### Esports and traditional sports participation

In addition, we compared the sports participation of the 633 esports players involved in the survey with that of 916 non-esports players. As shown in Table 5, it is found that esports players significantly outpace non-esports players in terms of sports participation. Besides, among players of different genres of esports games, those who play sports games and fighting games participate in sports activities significantly more than players of other game types. Additionally, as shown in Table 6, according to the results of the Mann-Whitney U test, there is no significant difference in the frequency of participation in traditional sports between esports players and non-esports players (*p*>0.05). However, among the traditional sports players in both groups, there is a significant difference in participation frequency between male and female participants (*p*<0.05), with a higher number of males engaging in frequent exercise compared to females.

**Table 5:** Comparison in sports participants between esports and non-esports players.

	Esports players	Non-esports players	χ^2^	*p*
Age	18.8 ± 1.5	18.6 ± 1.5		
Active in Traditional Sports	418 (355)	451 (514)	42.893	***p <0.001
Inactive in Traditional Sports	215 (278)	465 (402)		

Values in parentheses are expected counts; * *p*<0.05, ** *p*<0.01

*** *p*<0.001.

**Table 6:** Comparison in sports frequency between esports and non-esports players.

Traditional sports frequency	Esports players		Non-esports players		*p*
	Male (%)	Female (%)	Male (%)	Female (%)	
Almost everyday	19 (6.7)	7 (5.3)	9 (6.8)	14 (4.4)	0.67
Three to five times a week	63 (22.1)	27 (20.3)	32 (24.1)	59 (18.6)	
Once or twice a week	121 (42.5)	43 (32.3)	57 (42.9)	134 (42.1)	
Once or twice a month	62 (21.8)	35 (26.3)	24 (18)	75 (23.6)	
Less than a month	20 (7)	21 (15.8)	11 (8.3)	36 (11.3)	
*p*	*0.02		*0.03		

* *p*<0.05, ** *p*<0.01, *** *p*<0.001.

## Discussion

This study investigated the association between esports performance and traditional sports participation. The data analysis revealed significant differences among the three game categories: MOBA (PC), MOBA (Mobile), and FPS. Specifically, FPS players who participated in physical sports demonstrated significantly higher esports performance compared to those who did not engage in such activities. This finding provides preliminary evidence for incorporating traditional physical training into the regimen of FPS esports players in the future. In contrast, no significant difference was observed between participants in the other two categories. This discrepancy may be attributed to Deleuze’s assertion that FPS games demand more from players in terms of reaction time compared to MOBAs, with reaction speed being a critical skill in FPS games [53–55]. Physical exercise is known to enhance reaction capabilities [28,56–58], suggesting that FPS players who engage in physical sports may exhibit superior gaming skills and performance. Conversely, MOBA games require a comprehensive understanding of game dynamics and strategic planning, such as team compositions and coordination [59,60]. In MOBAs, tactical acumen significantly impacts multiplayer engagements and outcomes, surpassing the influence of mere operational skills [61]. Therefore, multiple factors can affect overall performance in MOBAs, making the impact of reaction time comparatively less significant.

Additionally, the data analysis indicated a unique trend in Honor of Kings: unlike in the other game categories, the duration a player has spent in the game correlated with their in-game ranking. This correlation may be due to the rank protection and reward mechanisms in Honor of Kings. When a player reaches a certain rank, they will not decrease in rank due to game failures and poor performance, and when they achieve consecutive victories, they can get a faster rank promotion. And Honor of Kings’ matchmaking system also tends to compensate players who have experienced consecutive losses. These mechanisms are different from that of LOL and PUBG. It can lead to players achieving higher ranks if they invest enough time in match play, which, to some extent, affects the objective evaluation of a player’s game performance based on rank in the game. In League of Legends, each rank promotion requires multiple promotion matches, which makes the promotion more difficult, and this tests the player’s skill level even more. Accordingly, in future research on esports performance, the measurement of performance across different games needs to be more targeted and further standardized.

In this study, no significant differences in esports performance were observed between participants engaged in competitive versus non-competitive sports or between those in individual versus team sports. Given the comprehensive physical fitness and athletic ability demands across the various types of sports included, and due to the high degree of similarity among some sports coupled with the diversity of sports types examined, it was challenging to analyze the specific relationship between particular physical abilities or training methods and esports performance. Additionally, data on the frequency, years of participation, and duration of physical exercise were collected from participants, and a correlation analysis was conducted with the players’ gaming performance. However, the results of the Gamma test (p>0.05) indicated no significant correlation between esports performance and the frequency, years, or duration of sports participation. This underscores the need for further multi-method research to determine which specific types of physical training or training modes are most effective for integration into esports training. Nevertheless, it is anticipated that physical exercise would positively impact esports performance.

Furthermore, our study revealed a notable trend: when contrasted with non-esports participants, individuals involved in esports exhibit a heightened level of engagement in physical sports activities. This is particularly pronounced among those who partake in sports and fighting games. These findings lend additional credence to the hypothesis that participation in esports may serve to enhance motivation toward physical sports involvement [62,63]. Additionally, according to previous literature, professional players with higher levels of skill in esports often demonstrate greater motivation and participation in physical exercise [64,65]. This trend also demonstrates a potential win-win relationship formed by participation in both esports and traditional sports. Reconciling the reciprocal benefits of physical exercise and esports participation represents a significant direction for future inquiry within the esports research field. Realizing this symbiotic relationship requires further empirical evidence and practical applications. These efforts could facilitate the integration of the respective strengths of physical exercise and esports, thereby maximizing their potential benefits and establishing a virtuous cycle. The implications of these findings are substantial and far-reaching for all stakeholders, including professional esports athletes, casual esports players, and non-esports participants alike.

## Limitation

The sample size of this study was relatively small, and some esports players in games such as RTS, strategy card games, and sports games were not included in the analysis because the number of such gamers is limited in this study.

Although ranking in games can provide a comprehensive and direct reflection of participants’ esports level, there are differences in the rank system and matchmaking system mechanisms between different games. Additionally, some rank systems may be influenced by factors such as game experience or matchmaking mechanisms, as highlighted in this study. Therefore, a more scientific model of esports performance is needed for future analysis to make research more scientifically sound.

Due to the wide range of sports types participants engage in, the sample size was insufficient to discuss players’ esports performance in each subcategory of sports specifically. It was not easy to conduct a targeted analysis of the differences in esports performance of each sports category or to explore the relationship between specific sports skills or physical fitness and esports performance. Further research with a more targeted approach and a larger sample size is needed for a more in-depth analysis.

## Conclusion

The results of this study indicate that players of FPS who engage in traditional sports demonstrate superior esports performance compared to those who do not. These findings provide preliminary evidence supporting the efficacy of integrating physical exercise with gameplay in esports training, which has the potential to enhance both the physical health and esports performance of athletes. However, the study did not reveal any significant associations between esports performance and either the duration of participation in esports games or the type of sports activities in which the participants engaged. This may be due to the comprehensive and overlapping physical fitness and athletic ability requirements of the included sports types, as well as the broad range of sports categories analyzed, which limited the ability to discern specific relationships between particular physical abilities and esports performance. Notably, the study did not identify any significant correlations between esports performance and sports frequency, duration, or years of engagement. These findings highlight the need for further multi-method research to investigate which specific types of physical training and modalities are most suitable for integration into esports training programs. In addition, our study underscores a key relationship: esports participation correlates with increased engagement in physical sports, especially in sports and fighting game genres. The task ahead lies in harnessing the symbiotic benefits of physical exercise and esports, a process that requires further evidence and practical applications. Ultimately, this win-win amalgamation of physical sports and esports could maximize their respective strengths and benefits for all involved.

## Theoretical and practical implications

The results of this study suggest that participation in traditional sports may confer a competitive advantage in esports, particularly within the FPS genre. This implies that the physical fitness and cognitive benefits derived from traditional sports could translate into enhanced performance in virtual environments. Consequently, esports training programs could benefit from incorporating structured physical exercise regimes, potentially improving players’ in-game performance and overall physical well-being. This integrative approach could reshape training paradigms in esports, emphasizing the holistic development of esports athletes.

The absence of significant associations between esports performance and duration or types of sports indicates that merely engaging in physical exercise may not be sufficient to predict or enhance esports performance. Instead, the quality, specificity, and perhaps the intensity of physical training might play more critical roles. This underscores the importance of designing tailored physical exercise programs that align with the specific demands of esports.

### Future research

Future research is needed to address the following aspects:

**Identification of specific training types:** To determine which types of physical training are most beneficial, research should aim to identify the physical and cognitive demands specific to different esports genres. Subsequent studies could then match these demands with targeted exercise interventions.

**Longitudinal studies:** Long-term studies are necessary to examine the sustained effects of physical exercise on esports performance. This would help in understanding whether short-term benefits translate into long-term improvements.

**Experimental designs:** Experimental studies that manipulate the type, frequency, and intensity of physical exercise could provide causal evidence for the benefits of integrating sports training into esports.

**Diverse esports genres:** Expanding the research to include various esports genres beyond FPS games would provide a more comprehensive understanding of the potential benefits of physical exercise across different competitive platforms.

**Practical applications**: Practical implications for the findings need to be explored through the development and testing of exercise programs within the esports community. This involves collaboration with esports organizations, coaches, and players to create and implement evidence-based training protocols.

## Supporting information

S1 File Approved Research Ethics Certificate (1). (PDF)

S2 File Approved Research Ethics Certificate (2). (PDF)

S3 File STROBE checklist combined PlosMedicine. (DOCX)

S4 File PLOSOne human subjects research checklist. (DOCX)

## Abbreviations

AOV: Arena of Valor
FPS: First Person Shooter
HOK: Honor of Kings
LOL: League of Legends
MOBA: Multiplayer Online Battle Arena
RTS: Real-Time Strategy
PUBG: Player Unknown’s Battlegrounds
