# Supplementary material for: Synergistic fields: Unveiling the potential win-win relationship between esports performance and traditional sports participation
Source: PLoS One. 2024 Aug 12;19(8):e0305880. doi: 10.1371/journal.pone.0305880 (PMC11318873; doi:10.1371/journal.pone.0305880)
Supplement: S1 File — (PDF) [file pone.0305880.s001.pdf]

香港中文大學
The Chinese University of Hong Kong

2022-01-12

Mr. TANG, Di
PhD Education - Faculty of Education

Survey and Behavioural Research Ethics
Reference No. SBRE-21-0432

Dear Mr. TANG, Di,

I write to inform you that the Survey and Behavioural Research Ethics Committee has granted approval in principle for you to conduct the surveys or observation of human behaviour by non-clinical means as declared in the application for the following research:

Principal Investigator:
Mr. TANG, Di

Affiliated Department/Unit:
PhD Education - Faculty of Education

Project Title:
The Relationship Between eSports Performance and Physical Ability

Name of Grant/Funding (Reference No.):

Kindly be reminded that you should also obtain approval from other research ethics committees within the University (e.g., Clinical Research Ethics Committee, Animal Experimentation Ethics Committee) if any parts of your research do not fall under the scope of our Committee. Thank you for your attention.

Yours Sincerely,

Ms. Eva Yeung
Secretary of Survey and Behavioural Research Ethics Committee
