# Supplementary material for: Synergistic fields: Unveiling the potential win-win relationship between esports performance and traditional sports participation
Source: PLoS One. 2024 Aug 12;19(8):e0305880. doi: 10.1371/journal.pone.0305880 (PMC11318873; doi:10.1371/journal.pone.0305880)
Supplement: S2 File — (PDF) [file pone.0305880.s002.pdf]

**Application Reference No.**	SBRE-21-0432
**Application Status**	Application Approved

PI and Project Information

**Principal Investigator (PI)**	TANG, Di
**Preferred Name of Principal Investigator (PI)**	Mr. TANG, Di
**PI Title / Position**	
**Survey and Behavioral Ethics Training Cert. Number**	S83905063 (Valid through 2026-12-18)
**Publication Ethics Training Cert. Number**	P98406333 (Valid through 2026-08-29)
**Supervisor(O365 Login ID)**	kwsum@cuhk.edu.hk (Professor SUM Kim Wai Raymond)
**Programme Code**	50027
**Programme Name**	PhD Education
**Department/Unit**	Faculty of Education
**Project Title**	The Relationship Between eSports Performance and Physical Ability
**Estimated Start Date of the Study**	2022-01-15
**Reason for Late Submission**	
**Estimated End Date of the Study**	2022-05-01
**Name of Grant/Funding (if applicable)**	
**Reference No. of Grant/Funding (if applicable)**	
**Amount Applying / Approved**	
**Status of Grant/Funding**	○ Approved<br>○ Pending<br>◉ Not Applicable
**A copy of your grant proposal (if applicable)**	

Summary of Research Proposal

Summarize, in layperson's language, the below details of the study with no more than 200 words per each:

(1) Objective(s)

(2) Significance

(3) Research Methodology(ies)

The research is to investigate if there is any relationship between the eSports performance and physical ability. The survey will collect some data about participants' eSports behaviors and their rank in the online game and their sports behaviors. At the end of the survey, we will ask participants whether they are willing to continue the further research. Those who are interested will receive a set of assessment on physical ability.

Participant Information Sheet or Consent Form

Questionnaires and measures

Type of Study

☐ This research will be conducted in established or commonly accepted educational settings, involving normal educational practices that are not likely to adversely impact students' opportunity to learn or assessment of educators. All participants are **adults**.

Type of Study	☐ This research will involve the use of educational tests (cognitive, diagnostic, aptitude, achievement), survey procedures, interview procedures or observation of public behaviour of **adult** participants. Recorded information cannot readily identify the participant (directly or indirectly/linked), OR any disclosure of responses outside of the research would NOT reasonably place participant at risk (criminal, civil liability, financial, employability, educational advancement, reputation).<br>☐ This research will involve benign behavioural interventions through verbal, written responses, (including data entry or audiovisual recording) from **adult** participants who agrees. Recorded information cannot readily identify the participant (directly or indirectly/linked), OR any disclosure of responses outside of the research would NOT reasonably place participant at risk (criminal, civil liability, financial, employability, educational advancement, reputation).<br>☐ This research is a secondary research project with identifiable information collected for some other initial activity, if information is publicly available, OR information is recorded in such a way that participant cannot readily be identified (directly or indirectly/linked); investigator does not contact participants and will not re-identify the participants.<br>☐ This is research on individual or group characteristics or behaviour (including, but not limited to, research on perception, cognition, motivation, identity, language, communication, cultural beliefs or practices, and social behaviour) or research employing survey, interview, oral history, focus group, program evaluation, human factors evaluation, or quality assurance methodologies. Recorded information cannot readily identify the participant (directly or indirectly/linked), OR any disclosure of responses outside of the research would NOT reasonably place participant at risk (criminal, civil liability, financial, employability, educational advancement, reputation). All participants are **adults**.<br>☑ Not applicable.

Participants

**Is there any participant involved?**	◉ Yes<br>○ No
**Number of Healthy Adults:**	500
**Number of Children (under 18 years old):**	0
**Number of Pregnant Women:**	0
**Number of Outpatients:**	0
**Number of Inpatients:**	0
**Number of Prisoners:**	0
**Number of Cognitively Impaired Persons, please specify**	0
**Others, please specify**	
**Total Number of Participants:**	500

Inclusion / Exclusion Criteria

Breakdown of the planned number of participants

**Number of Male Adult:**	250
**Number of Female Adult:**	250
**Number of Children:**	0
**Lower age limit of the participants (0 to indicate no limit):**	18
**Upper age limit of the participants (999 to indicate no limit):**	999
**Are there any participant recruitment restrictions based on race/ethnicity of the participant?**	○ Yes<br>◉ No

What are the inclusion criteria in participant recruitment?

Healthy University Students in CUHK

What are the exclusion criteria in participant recruitment?

Students who are unable to finish the questionnaire

Are the participants in a dependent relationship with the researchers (e.g., researchers' children or students)

- ○ Yes, please provide details:
- ◉ No

Participant Recruitment

Describe how the participants will be recruited with no more than 100 words

The questionnaire will be created online and a QR code/electronic link will be present on the website of Physical Education Unit of The Chinese University of Hong Kong (we will ask PEU for collaboration and permission in advance). And also we will send QR code or link to students who are willing to take part in the research after PE courses

---

Advertisement used to recruit participants for your research (if applicable)

Tasks / Activities Performed by the Participants

Task 1 of 2

Description of task / activity to be included

We will ask participants who finished previous questionnaire if they are willing to take part in this physical ability assessment. The some assessment will be conducted based on the part of physical competence of Canadian Assessment of Physical Literacy.

**Target participants of this task / activity**	University students in CUHK	
**Duration of participants to be involved in this task / activity**	30	minutes
**Indicate the number of times / research visits the participant needs to make to complete this task / activity**	1visits	
**Questionnaires and measures that will be used in the study**	assessment manual.pdf (3.41MB)	

Risk Level

- ◉ Minimal risk
- ○ More than minimal risk (describe the risk)

Risk Description

Because the assessment of physical ability involves some physical movement so there might be possibility of sports injury. But we will set all the physical activity at a medium intensity. Only healthy adults will be recruited in this test (health condition will be confirmed on a questionnaire before the test). And participants can quit the test anytime if they feel uncomfortable, All the test will be conducted with the guidance and assistance of a professional fitness coach onsite.

The PI is responsible for ensuring that all research participants give informed consent before enrolling into the research.

Do you request a waiver of informed consent?

- ○ Yes
- ◉ No

Please upload Participant Information Sheet or Consent Form. Consent Form.docx (0.02MB)

Please upload Assent Form (if applicable).

Task 2 of 2

Description of task / activity to be included

an online questionnaire to investigate participants' eSports and traditional sports behaviors

**Target participants of this task / activity**	CUHK university students	
**Duration of participants to be involved in this task / activity**	20	minutes
**Indicate the number of times / research visits the participant needs to make to complete this task / activity**	1visits	
**Questionnaires and measures that will be used in the study**	問卷_繁體字版.docx (0.05MB)	
**Risk Level**	◉ Minimal risk<br>○ More than minimal risk (describe the risk)	

Risk Description

The questionnaire is only to collect some information about participants' daily behavior (sports and eSports habits) so it won't result in any physical or mental harm or discomfort in the course. All the participants can quit the study anytime if they feel uncomfortable.

The PI is responsible for ensuring that all research participants give informed consent before enrolling into the research.

**Do you request a waiver of informed consent?**	○ Yes<br>◉ No
**Please upload Participant Information Sheet or Consent Form.**	Consent Form.docx (0.02MB)
**Please upload Assent Form (if applicable).**	

Remuneration to Participant

**Participant will be**	○ Reimbursed, HKD<br>◉ Not Reimbursed<br>○ Others, please specify:
**Do you have any potential conflicts of financial interest to declare?**	◉ No<br>○ Yes, please specify:

Data Storage and Access

Where will the data be stored?

All the data collected will be stored in a personal computer with a password of the investigator only.

Who will have access to the data?

Only the investigator can have access to the data.

What will happen to the data after research is completed?

All the data will be erased from the computer of the research is completed.

**Will you collect data on audiotape, film/video, or other electronic media?**	No

Deception

Does your research involve the use of deception? | No

Debriefing

Participants must be fully debriefed about the purpose of the research at the end of the study. A written debriefing handout must be provided to the participants.

Debriefing handout | Debriefing Form.doc (0.03MB)

Declaration

Declaration | I confirm that the information above is to the best of my knowledge accurate. I shall take reasonable care to ensure that the project is conducted in accordance with the Guidelines for Survey and Behavioural Research Ethics. I will obtain approval from other responsible units within CUHK (e.g. The Joint Chinese University of Hong Kong (CUHK) Hospital Authority New Territories East Cluster (NTEC) Clinical Research Ethics Committee (CREC), Animal Experimentation Ethics Committee (AEEC), University Safety Office/University Laboratory Safety Office) where appropriate.

Reviewer Assessment Checklist

1 Risks to participants are minimized; research design does not unnecessarily expose subjects to risk.

1 Risks to participants are reasonable in relation to anticipated benefits, if any, to subjects, and the importance of the knowledge that may reasonably be expected to result.

1 Selection of subjects is equitable.

1 Informed consent will be sought (or can be waived) from each prospective subject or the subject's legally authorized representative, and informed consent will be appropriately documented.

1 There are adequate provisions to protect the privacy of participants and to maintain the confidentiality of data.

Status Log

Status	On	By (Role)	Remarks
Application Approved	2022-01-12 12:09	Secretary of SBREC	
Approved by First Reviewer	2022-01-08 11:57	1st Reviewer	The research study is in line with the prevailing SB research ethics.
Under Review	2022-01-07 15:11	Secretary of Faculty Sub-committee	
Under Vetting	2022-01-07 15:11	Secretary of Faculty Sub-committee	
Submitted for Review	2022-01-05 14:12	Supervisor	
Reviewing by Supervisor	2022-01-05 14:04	Applicant	
Return for Revision	2022-01-04 17:30	Secretary of Faculty Sub-committee	Dear Di,<br><br>Please consider the following items for your SBRE application:<br>- For the SBRE application under "Inclusion / Exclusion Criteria", please input "999" for upper age limit of the participants if there is no limit<br>- For the consent form under "Statement of Consent", please indicate the "Duration of information kept by the researcher for the study"<br><br>Thank you.
Submitted for Review	2021-12-30 15:57	Supervisor	

Reviewing by Supervisor	2021-12-30 15:28	Applicant	
Draft	2021-12-30 12:26	Applicant	
